# Vitamin D deficiency down-regulates Notch pathway contributing to skeletal muscle atrophy in old wistar rats

**DOI:** 10.1186/1743-7075-11-47

**Published:** 2014-09-30

**Authors:** Carla Domingues-Faria, Audrey Chanet, Jérôme Salles, Alexandre Berry, Christophe Giraudet, Véronique Patrac, Philippe Denis, Katia Bouton, Nicolas Goncalves-Mendes, Marie-Paule Vasson, Yves Boirie, Stéphane Walrand

**Affiliations:** Université d’Auvergne, Unité de Nutrition Humaine, Equipe ECREIN, CLARA, CRNH Auvergne; INRA, UMR 1019, UNH, CRNH Auvergne, Clermont Université, 63000 Clermont-Ferrand, France; Université d’Auvergne, Unité de Nutrition Humaine, Equipe NuTriM, CRNH Auvergne; INRA, UMR 1019, UNH, CRNH Auvergne, Clermont Université, 63000 Clermont-Ferrand, France; Université d’Auvergne, Unité de Nutrition Humaine, Installation Expérimentale de Nutrition, CRNH Auvergne; INRA, UMR 1019, UNH, CRNH Auvergne, Clermont Université, 63000 Clermont-Ferrand, France; INRA, UMR1019, UNH, CRNH Auvergne, 63000 Clermont-Ferrand, France; Centre Jean Perrin, Unité de Nutrition, 63000 Clermont-Ferrand, France; CHU Clermont-Ferrand, Service de Nutrition Clinique, 63003 Clermont-Ferrand, France

**Keywords:** Vitamin D deficiency, Aging, Skeletal muscle atrophy, Notch signalling

## Abstract

**Background:**

The diminished ability of aged muscle to self-repair is a factor behind sarcopenia and contributes to muscle atrophy. Muscle repair depends on satellite cells whose pool size is diminished with aging. A reduction in Notch pathway activity may explain the age-related decrease in satellite cell proliferation, as this pathway has been implicated in satellite cell self-renewal. Skeletal muscle is a target of vitamin D which modulates muscle cell proliferation and differentiation *in vitro* and stimulates muscle regeneration *in vivo*. Vitamin D status is positively correlated to muscle strength/function, and elderly populations develop a vitamin D deficiency. The aim of this study was to evaluate how vitamin D deficiency induces skeletal muscle atrophy in old rats through a reduction in Notch pathway activity and proliferation potential in muscle.

**Methods:**

15-month-old male rats were vitamin D-depleted or not (control) for 9 months (n = 10 per group). Rats were 24-month-old at the end of the experiment. Gene and/or protein expression of markers of proliferation, or modulating proliferation, and of Notch signalling pathway were studied in the *tibialis anterior* muscle by qPCR and western blot. An unpaired student’s *t*-test was performed to test the effect of the experimental conditions.

**Results:**

Vitamin D depletion led to a drop in concentrations of plasma 25-hydroxyvitamin D in depleted rats compared to controls (-74%, p < 0.01). *Tibialis anterior* weight was decreased in D-depleted rats (-25%, p < 0.05). The D-depleted group showed -39%, -31% drops in expression of two markers known to modulate proliferation (Bmp4, Fgf-2 mRNA levels) and -56% drop in one marker of cell proliferation (PCNA protein expression) compared to controls (p < 0.05). Notch pathway activity was blunted in *tibialis anterior* of D-depleted rats compared to controls, seen as a down-regulation of cleaved Notch (-53%, p < 0.05) and its target Hes1 (-35%, p < 0.05).

**Conclusions:**

A 9-month vitamin D depletion induced vitamin D deficiency in old rats. Vitamin D depletion induces skeletal muscle atrophy in old rats through a reduction in Notch pathway activity and proliferation potential. Vitamin D deficiency could aggravate the age-related decrease in muscle regeneration capacity.

**Electronic supplementary material:**

The online version of this article (doi:10.1186/1743-7075-11-47) contains supplementary material, which is available to authorized users.

## Introduction

One of the most striking effects of ageing is an involuntary loss of muscle mass known as sarcopenia. The development of sarcopenia appears to be multifactorial and includes anabolic resistance to dietary amino acids, hormonal changes and sedentary lifestyle [1]. The diminished ability of aged muscle to self-repair is also a key factor behind sarcopenia [2–4]. Muscle loss during ageing may partly depend on the accumulation of repeated episodes of incomplete repair and regeneration throughout the life span following overt injury but also daily small damages that may not be perceived *via* pain or alteration in function [5]. Muscle repair occurs in 4 interdependent phases: (1) degeneration; (2) inflammation; (3) regeneration, involving satellite cells (SC) that enter the cell cycle and differentiate to form newly multinucleated cells or to repair surviving fibers; (4) remodelling and repair [6]. This process is thus reliant on SC located underneath basal lamina of myofibers [7, 8]. SC pool size shrinks significantly with ageing [9]. Shefer et al. [10] showed that number of SC cells per freshly-isolated mice myofiber declines with age whereas SC differentiation potential remains unchanged [10]. However, the state of SC pool with ageing is controversial because some investigators have demonstrated that although no changes occur in the SC number with ageing, their physiological function, i.e. regenerative potential, was impaired [11–13].

As recently revealed, the hypothesis of a decreased SC proliferative capacity with age can also be explained by an age-related decrease in Notch pathway activity [14]. Notch is a highly conserved transmembrane receptor whose pathway plays a central role in muscle development and regeneration [15–17]. Binding of the Notch ligand, e.g. transmembrane protein Delta-1, promotes two proteolytic cleavage events [18]. First, an ADAM (A disintegrin and metalloprotease domain) protease cleaves Notch receptor to generate the transmembrane fragment Notch (^TM^Notch) [19]. Second, a γ-secretase complex cleaves ^TM^Notch [20], leading to the release of the intracellular domain of Notch receptor (^Nicd^Notch). ^Nicd^Notch then translocates to the nucleus where it acts as a transcription factor to promote the transcription of its target genes, such as Hes1 or Hey1 [21], which are implicated in the blockade of cell differentiation and the maintenance of cell self-renewal [22–25]. Mutant mice expressing the Notch inhibitor dnMAML1-gfp in muscle stem cells show smaller muscles and fewer SC [26]. The decline of Notch pathway activity with ageing may in part explain the reduced number of SC able to regenerate muscle cells [27]. Although changes occur in SC cells during aging, environmental factors still play a significant role in muscle regeneration [28].

Observational studies have shown that vitamin D status is positively correlated to muscle strength and function [29]. Vitamin D is derived from the action of ultraviolet (UV) light on skin and from diet [30, 31]. Once produced in skin or absorbed by the gut, vitamin D is carried in blood, mostly by vitamin D-binding protein, to the liver where it undergoes 25-hydroxylation to form calcifediol (25(OH) D), the major circulating metabolite of vitamin D [32]. Vitamin D input is largely reflected by blood 25(OH) D concentrations, and blood 25(OH) D is widely used as a measure of vitamin D status. 25(OH) D undergoes a last hydroxylation step by 1-α-hydroxylase enzyme (CYP27B1), expressed in kidney and many other tissues, to form the active hormone 1,25-dihydroxyvitamin D (1,25(OH)_2_D or calcitriol) [33]. Vitamin D plays a role in numerous physiological processes through both genomic and non-genomic effects [33, 34]. Note that the various actions of vitamin D can be dependent on or independent of its binding on the nuclear receptor VDR (vitamin D receptor), as shown by previous studies in myotubes or osteoblasts [35, 36].

Skeletal muscle is a target of vitamin D [34], and several *in vitro* and *in vivo* studies have been led to analyze its effects on muscle. *In vitro* studies show that vitamin D modulates muscle cell proliferation and differentiation [35, 37, 38]. *In vivo*, vitamin D injections in adult rats promote cell proliferation and consequently the regenerative process in skeletal muscle after a crush injury [39]. Besides its key role in muscle cell proliferation and differentiation, vitamin D also regulates muscle contractile function [40, 41]. Whole together, these data demonstrate the necessity of vitamin D for the maintenance of structural integrity and function of skeletal muscle.

Vitamin D deficiency is common in elderly populations and has been associated with muscle weakness [42]. However, an *in vivo* study has highlighted that muscle weakness was rather a consequence of hypophosphatemia associated with hypovitaminosis D [43]. Furthermore, VDR expression in human muscle tissue decreases with age [44].

Bone morphogenetic protein 4 (Bmp4) and fibroblast growth factor-2 (Fgf-2) are two controllers known to be involved in the modulation of muscle cell proliferation and differentiation. Bmp4 regulates the transition from proliferation to differentiation [45], and Fgf-2 enhances the number of SC held in a proliferative state without suppressing the transition to the state of differentiation [46]. Interestingly, previous studies have shown that vitamin D modulates Bmp4 and FgF-2 expression [47, 48], and Notch pathway and Bmp4 interact to control proliferation/differentiation transition [49].

Prompted by these previous investigations, we addressed the hypothesis that vitamin D deficiency in old rats reduces the potential of skeletal muscle to regenerate by down-regulating the activity of the Notch signalling pathway, leading to muscle atrophy. After 9 months of vitamin D depletion, we studied the expression of markers implicated in the Notch pathway activity and in the modulation of proliferation in skeletal muscles in 24-month-old rats. We found that 9-month of vitamin D depletion induced a significant vitamin D deficiency in old rats and led to skeletal muscle atrophy, due at least in part to a reduced Notch pathway activity which controls muscle cell proliferation.

## Material and methods

### Ethics statement

All animal procedures were approved by the institution’s animal welfare committee (Comité d’Ethique en Matière d’Expérimentation Animale Auvergne: CEMEAA; Permit number: CE 93–12) and were conducted in accordance with the European’s guidelines for the care and use of laboratory animals (2010-63UE). Animals were housed in the animal facility of the INRA Research for Human Nutrition (Agreement N°: C6334514). Rats were purchased from JANVIER (Le Genest St Isle, France). At the end of the experiment, the rats were sacrificed by decapitation after isoflurane anaesthesia and all efforts were made to minimize animal suffering.

### Experimental protocol

Male 15-month-old Wistar rats were housed in a temperature- (22 ± 0.8°C) and humidity-controlled room, maintained on a 12 h light/dark cycle and given *ad libitum* access to standard chow and water for a 2-week acclimatization. The rats were then randomly assigned (n = 10 per group) to either the AIN-93 M maintenance diet or to a modified AIN-93 M diet with no vitamin D (TestDiet, Missouri, USA) for 9 months. Table 1 lists the diet compositions. Rats fed with the vitamin D-depleted diet were also housed under UV-filtered lamps (OSRAM, France) avoiding any vitamin D epidermal synthesis. Food intake was recorded every two weeks and records were stopped two weeks before the end of the experiment. Body weight was recorded weekly throughout the experiment. Rats were fasted (16 h) at the time of euthanasia. Animals were 24-months old on the day of euthanasia. At the end of the experiment, the rats were weighed then sacrificed by decapitation after isoflurane anaesthesia, and the *tibialis anterior* (TA) and *soleus* muscles were rapidly removed from both hind limbs and weighed. Muscle samples were quickly frozen in liquid nitrogen and stored at -80°C until analysis.

**Table 1:** **Composition of maintenance diet and vitamin-D-depleted diet**

%	AIN93M maintenance diet	AIN93M diet without vitamin D
**Carbohydrates**	73	73
**Protein** (free of vitamin D)	13	13
**Fat**	4.1	4.1
**Fiber**	5	5
**Vitamin and mineral mix**	4.9*	4.9**

*containing 1 IU/g of vitamin D3, **without vitamin D3.

Animals that died during the experiment or developed tumors or renal insufficiencies were excluded from the analysis.

### Body composition analysis

Control and vitamin D-depleted rats were subjected to magnetic resonance imaging (MRI) using Echo MRI (Echo Medical Systems, Houston, TX) to determine body composition at the beginning and the end of the experimental period. Lean mass (LM) and fat mass (FM) were expressed as percentage of body weight.

### Measure of plasma vitamin D and serum calcium and phosphorus

Blood samples were collected into EDTA tubes (Venosafe®, Terumo, France) at the beginning and the end of the experimental procedure and centrifuged at 1300 *g* for 10 min at 4°C to separate the plasma which was then rapidly frozen in liquid nitrogen and stored at -80°C until analysis. Blood was also collected into dry tubes (Venosafe®, Terumo, France) following depletion period, and after an incubation for 20 min at room temperature they were centrifuged at 1300 *g* for 10 min at 4°C to separate the serum which was then rapidly frozen in liquid nitrogen and stored at -80°C until analysis.

Plasma 25(OH) D levels were measured using a 25-OH Vitamin D (direct) ELISA kit (PromoKine, France) according to the manufacturer’s instructions.

Serum calcium and phosphorus levels were measured using an automat Konelab 20 (Thermo Scientific, MA, United States).

### Quantitative RT-PCR analysis

Total RNA was extracted using Tri-Reagent according to the manufacturer’s instructions. RNA quality was checked by agarose gel electrophoresis. RNA quantity was measured by determining the absorbencies at 260 and 280 nm. The level of mRNAs corresponding to genes of interest was measured by reverse transcription followed by RT-PCR using a Rotor-Gene Q system (Qiagen, France). One μg of total RNA was reverse-transcribed using a RT^2^ First Strand Kit (Qiagen, France).

In order to analyse a panel of genes related to biological pathways (cellular structure and function, apoptosis, proliferation, metabolism, muscle differentiation, Notch pathway and regulation of anabolism), a RT^2^ Profiler Custom PCR Array was used to simultaneously examine the mRNA levels of genes of interest, including four housekeeping genes, in Rotor-disc 100 format according to the manufacturer’s protocol (SuperArray Bioscience Corporation) [50, 51]. mRNA expression for each target gene in control and vitamin D depleted samples was normalized using expression of Tbp as a housekeeping gene and was relative to control group according to the 2^-ΔΔCT^ method, as described previously [52].

### Western blot analysis

A 50-mg sample of TA muscle was lysed in an ice-cold lysis buffer (50 mM HEPES pH 7.4, 150 mM NaCl, 10 mM EDTA, 10 mM NaPPi, 25 mM β-glycerophosphate, 100 mM NaF, 2 mM Na orthovanadate, 10% glycerol, 1% Triton X-100, Sigma-Aldrich, MO, United States) containing 1:200 of protease-inhibitor cocktail (Sigma-Aldrich, MO, United States). Protein concentration was determined using a Micro BCA^TM^ Protein assay kit (Thermo Scientific, MA, United States). Prior to SDS-PAGE, proteins were dissolved in a denaturing buffer containing 0.02% Bromophenol blue and 20% 2-β-mercaptoethanol and heated for 5 min at 95°C. Protein expressions were measured by loading 50 μg of denatured proteins onto a polyacrylamide gel. SDS-PAGE-separated proteins were transferred to a polyvinylidene membrane (Millipore, Molsheim, France). Immunoblots were blocked with TBS-Tween-20 0.1% containing 5% bovine serum albumin (for detection of ^TM^Notch1) or 5% dry milk (for detection of the others proteins), then probed overnight at 4°C with primary antibodies. The following primary antibodies were used: anti-VDR (1:1000; EPITOMICS; ref 3277–1), anti-proliferating cell nuclear antigen (anti-PCNA; 1:1000; Sigma; P8825), anti-Delta 1 (1:200; Santa Cruz Biotechnology; sc-9102), anti-transmembrane fragment Notch1 (anti-^TM^Notch1; 1:200; Santa Cruz Biotechnology; sc-6015), anti-p38 (1:10000; Sigma; M0800).

After several washes with TBS-Tween-20 0.1%, immunoblots were incubated with a horseradish peroxidase-conjugated secondary antibody for one hour at room temperature. The secondary antibody used was the horseradish peroxidase-conjugated anti-rabbit immunoglobulin (1:2000; Dako, P0399).

The immune-reactive strips were visualized by chemiluminescence (ECL Western Blotting Substrate, Pierce, IL). Luminescent secondary antibodies were visualized using MF ChemiBis 2.0 (DNR Bio-Imaging Systems, Israel). Intensity of the strips was quantified by densitometry using Multi Gauge V3.2 software (Fujifilm, Japan). Expression of the total amount of p38 was used to normalize protein loading between samples as previously described [53–56].

### Statistical analysis

All data are presented as means ± SEM. For food intake and body weight parameters, a repeated measures ANOVA was performed to test the conditions throughout the experiment. Concerning the others parameters studied, an unpaired student’s *t*-test was performed to test the effect of the experimental conditions. Statistical analysis was performed using StatView (version 4.02; Abacus Concepts, Berkeley, CA). Values of *p <* 0.05 (flagged *), or *p <* 0.001 (flagged **) were considered significant.

## Results

### Effect of vitamin D depletion on food intake and morphological parameters

**Figure 1:**
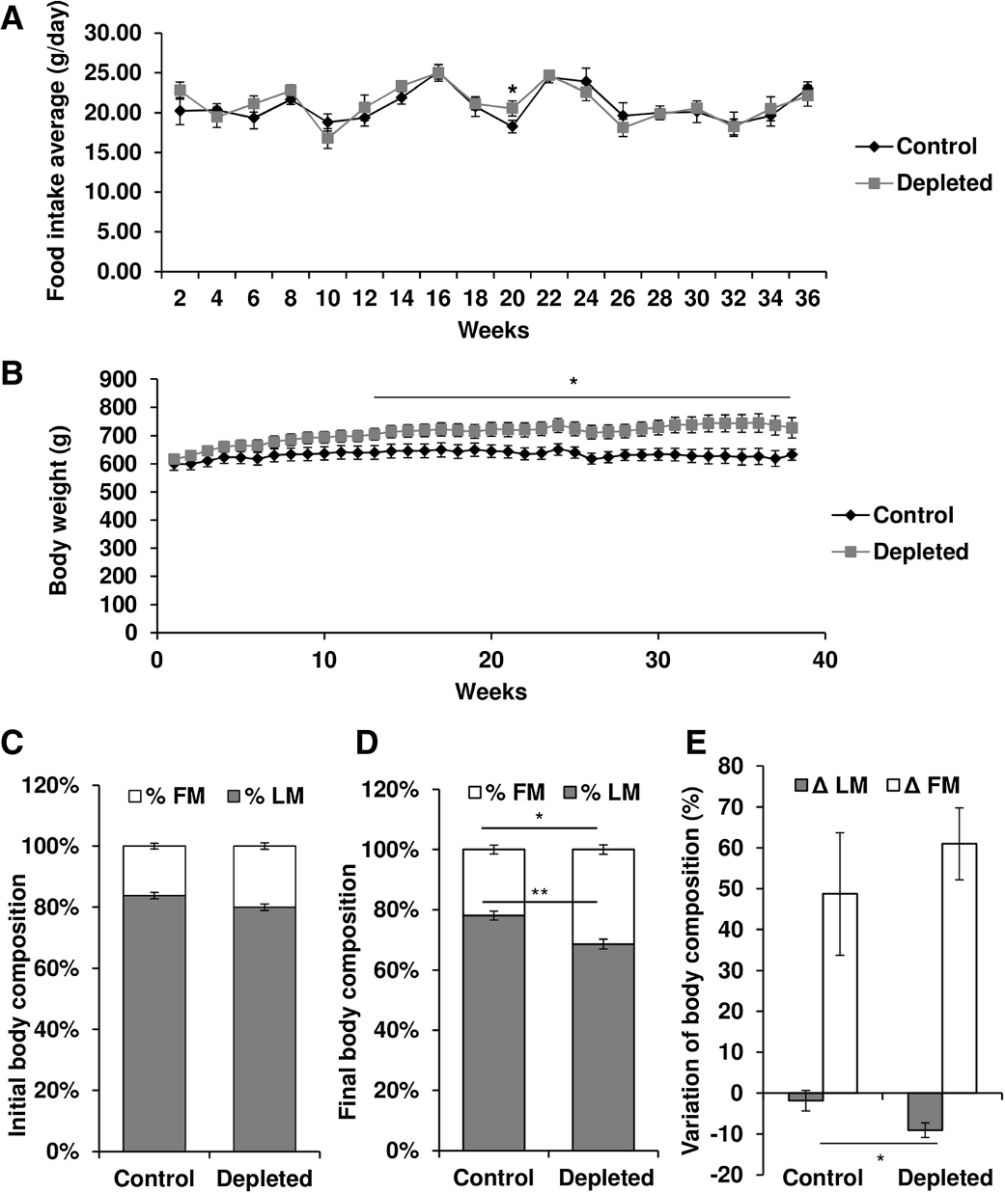
**Effect of vitamin D depletion on food intake and morphological parameters in old rats. (A)** Food intake of control and vitamin D-depleted rats throughout the experimental period (n = 7 for control group and n = 9 for depleted group). Food intake was similar between the two groups except at week 20. Data presented are means ± SEM; *p < 0.05. **(B)** Body weight was measured throughout the experiment (n = 7 for control group and n = 9 for depleted group). Body weight of vitamin D-depleted rats increased throughout the experiment and became significantly different in comparison with their control counterparts after 13 weeks. Data presented are means ± SEM; *p < 0.05. At the beginning **(C)** and the end **(D)** of the depletion period, rats from each group were subjected to magnetic resonance imaging (MRI) using Echo MRI to determine body composition. Fat mass (FM) and lean mass (LM) were expressed as percentage of body weight (n = 7 for control group and n = 9 for depleted group). At the beginning of the experiment, body composition of D-depleted and control rat was similar. In contrast, percent fat mass was significantly increased by 43% (p < 0.05) whereas percent lean mass was reduced by 12% (p < 0.001) in D-depleted rats compared to controls at the end of the experiment. Data presented are means ± SEM; *p < 0.05 and **p < 0.01. **(E)** Body composition of rats from each group was determined at the beginning and the end of the experimental period, allowing us to calculate variation in fat mass (Δ FM) and lean mass (Δ LM) throughout the experiment (n = 7 for control group and n = 9 for depleted group). The loss of LM was greater and the gain of FM tended to be more important in vitamin-D depleted rats compared to control rats. Data presented are means ± SEM; *p < 0.05.

**Figure 2:**
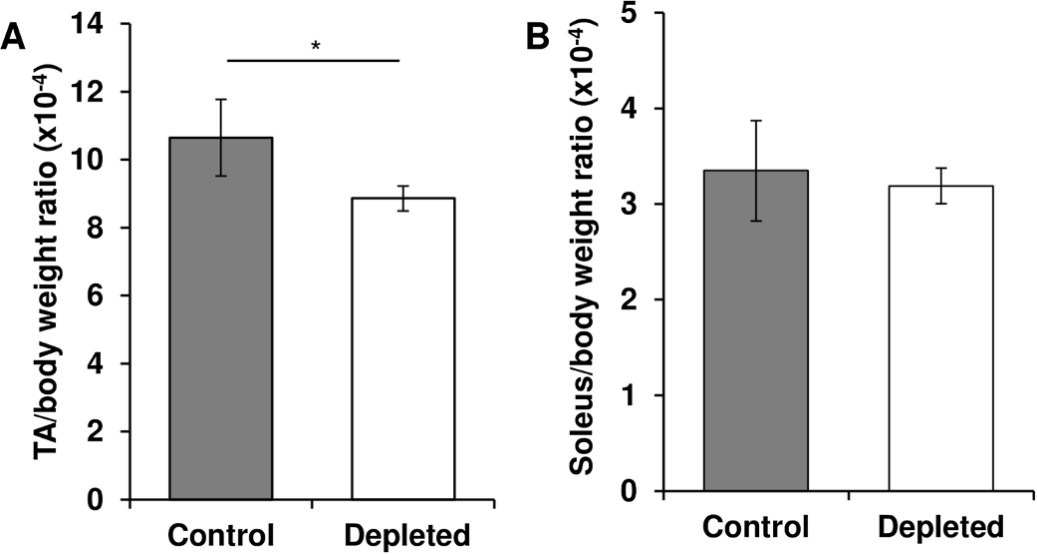
**Effect of vitamin D depletion on** ***tibialis anterior*** **or** ***soleus*** **/body weight ratio.** On the day of euthanasia, *tibialis anterior* (TA) **(A)** and *soleus* **(B)** of control and depleted rats were removed and weighed. TA or *soleus*/body weight ratio was calculated for each rat (n = 7 for control group and n = 9 for depleted group). TA/body weight ratio decreased in vitamin D-depleted old rats compared to control rats, whereas soleus/body weight ratio remained stable. Data presented are means ± SEM; *p < 0.05.

Mean daily food intake throughout the experiment was equivalent between the two diet groups, except at week 20, i.e. food intake of vitamin D-depleted rats was slightly but significantly more important than in the control group (Figure 1A). Body weight of vitamin D-depleted rats increased throughout the experiment and became significantly different in comparison with their control counterparts after 13 weeks (Figure 1B). At the beginning of the experiment, body composition of D-depleted and control rat was similar (Figure 1C). In contrast, percent fat mass was significantly increased by 43% (p < 0.05) whereas percent lean mass was reduced by 12% (p < 0.001) in D-depleted rats compared to controls at the end of the experiment (Figure 1D). Moreover, D-depleted rats showed significantly greater variation in lean mass over the experimental period than controls (control group *vs.* depleted group: -2% *vs.* -9%, p < 0.05; Figure 1E). Fat mass variation was similar between the two groups but tended to be higher in vitamin D-depleted rats. Type II muscle mass was preferentially affected by vitamin D depletion, since TA/body weight ratio decreased in vitamin D-depleted old rats (-25%, p < 0.05, Figure 2A), whereas type I soleus/body weight ratio remained unchanged between the two groups (Figure 2B).

### Effect of vitamin D depletion on vitamin D, calcium and phosphorus status and muscle VDR expression

**Figure 3:**
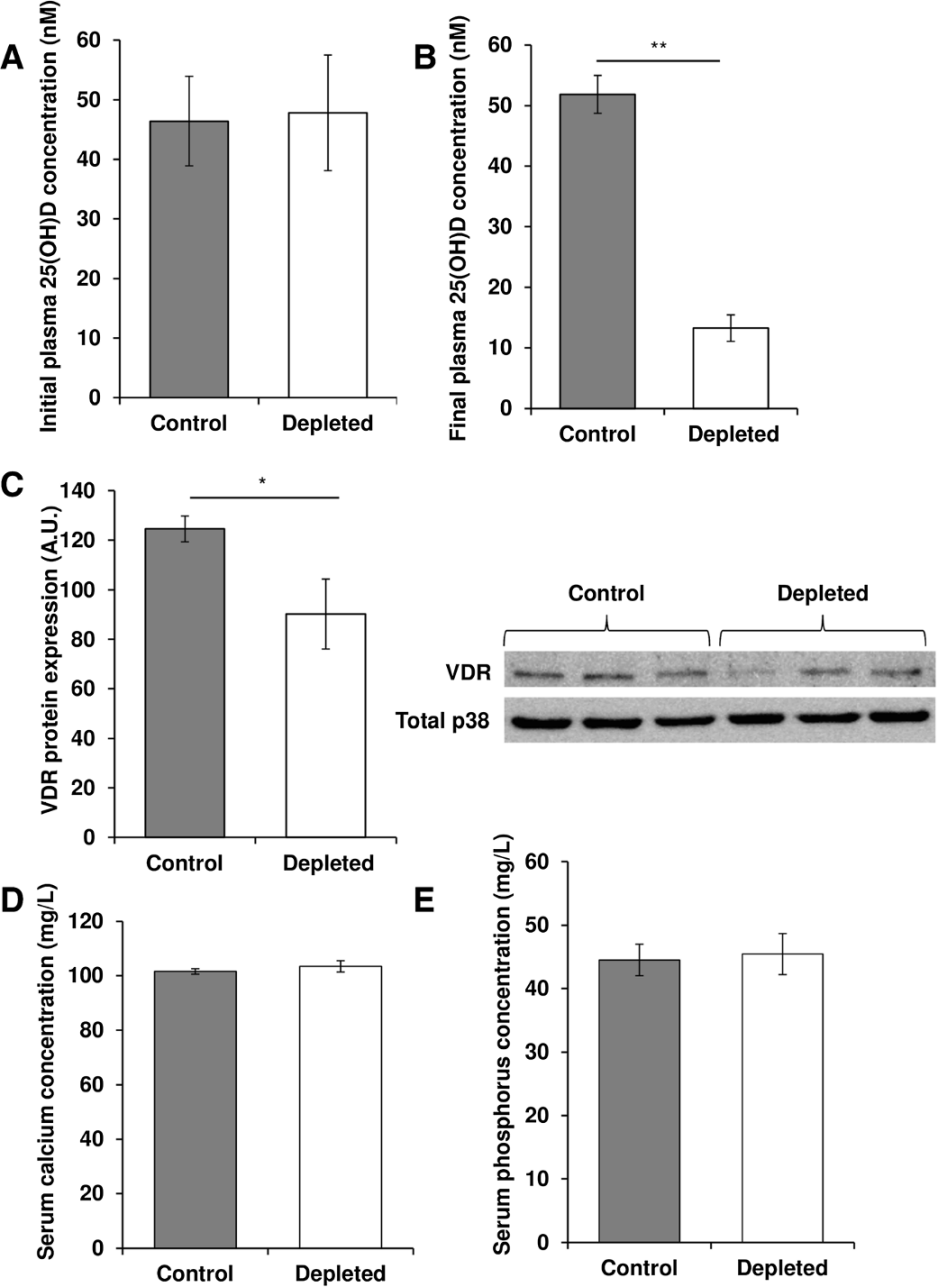
**Vitamin D, calcium and phosphorus status and muscle VDR expression in old rats.** At the beginning **(A)** and the end **(B)** of the experimental period, blood from control and depleted rats was collected and plasma was separated. Then, plasma 25(OH) D was measured using an ELISA kit according to the manufacturer’s instructions (n = 7 for control group and n = 9 for depleted group). 25(OH) D concentrations were similar at the beginning of the protocol and showed a severe drop in vitamin D status in depleted rats compared to controls after the depletion period. Data presented are means ± SEM; *p < 0.05 and **p < 0.01. **(C)** VDR protein expression was analyzed in muscle by western blotting and quantified using Multi Gauge V3.2 software. Expression of the total amount of p38 was used to normalize protein loading between samples (n = 7 for control group and n = 9 for depleted group). VDR protein expression was down-regulated in the skeletal muscle of the depleted group. A.U. = arbitrary units. Data presented are means ± SEM; *p < 0.05. At the end of the experiment, blood from control and depleted rats was collected and serum was separated. Then, serum calcium **(D)** and phosphorus **(E)** were measured using an automat Konelab 20 (n = 7 for control group and n = 9 for depleted group). Calcium and phosphorus concentrations were similar between control and depleted groups. Data presented are means ± SEM; *p < 0.05.

At the beginning of the experiment, blood 25(OH) D concentration was similar between the two groups (Figure 3A). As expected, a severe drop in vitamin D status was observed after 9 months of vitamin D depletion (-74%, p < 0.01, Figure 3B). Furthermore, VDR protein expression was down-regulated in the skeletal muscle of the depleted group (-28%, p < 0.05, Figure 3C).To ensure that muscle atrophy was not related to changes in blood calcium and phosphorus due to vitamin D depletion, serum calcium (Figure 3D) and phosphorus (Figure 3E) were measured and no differences were observed between the two groups.

### Effect of vitamin D depletion on expressions of proliferation and Notch pathway markers in *tibialis anterior*

In order to screen the putative pathways related to muscle mass changes with the vitamin D-depleted diet, we ran a targeted PCR array to study the expression of key genes regulating cell structure and function, apoptosis, metabolism, muscle differentiation and muscle anabolism, Notch pathway and cell proliferation.

mRNA expression of genes known to regulate apoptosis, metabolism, anabolism, myogenesis, and muscle cell structure, were impaired following vitamin D depletion (see Additional file 1).

Results also showed that the expression of the gene clusters related to Notch pathway and cell proliferation were significantly modulated by vitamin D depletion.

**Figure 4:**
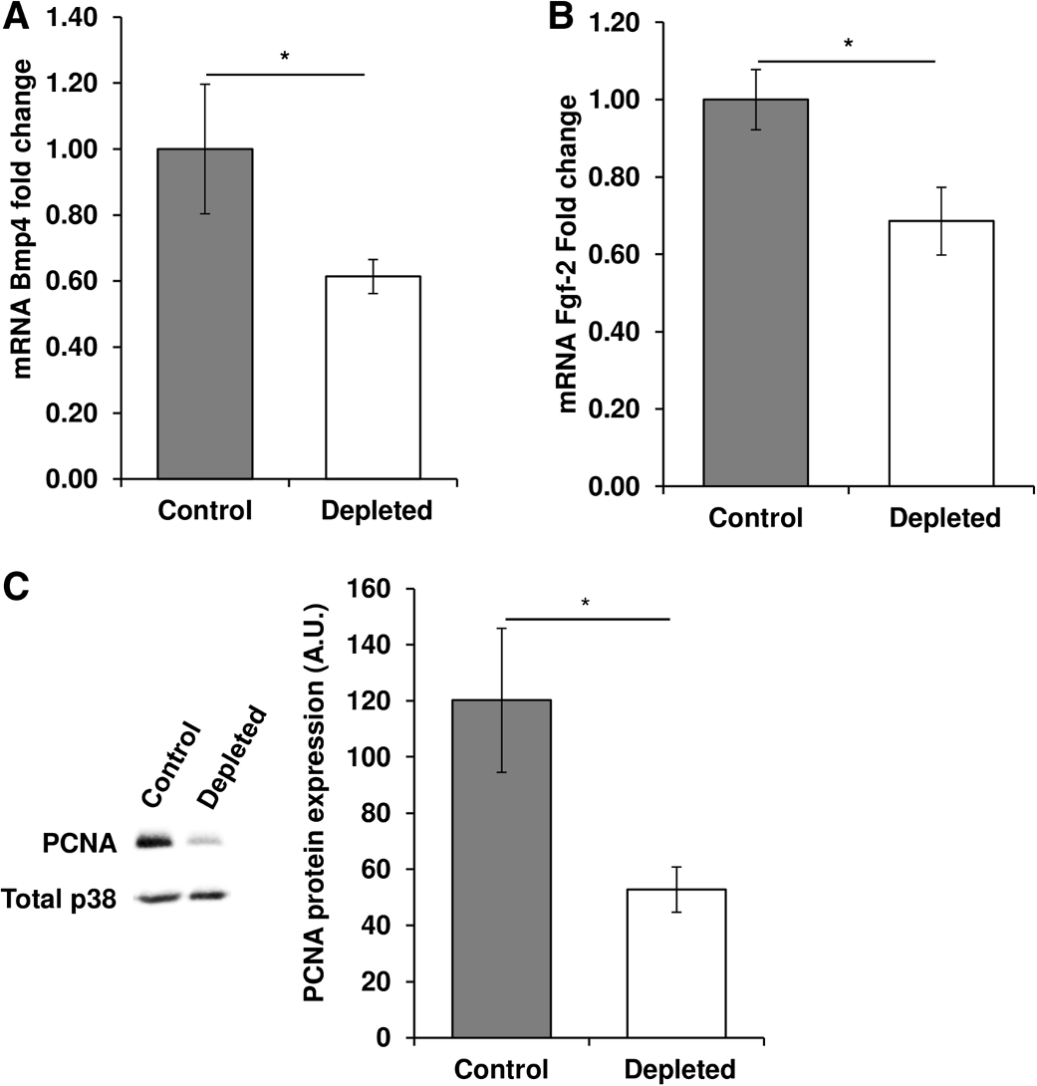
**Effect of vitamin D depletion on expression of proliferation markers and regulators in old rats. (A)** mRNA expression of Bmp4 and **(B)** Fgf-2 in *tibialis anterior* of control and depleted rats was analyzed using a RT^2^ Profiler Custom PCR Array following the manufacturer’s protocol. Bmp4 and Fgf-2 mRNA in control and vitamin D depleted samples was normalized using expression of Tbp as a housekeeping gene and was relative to control group according to the 2^-ΔΔCT^ method (n = 7 for control group and n = 9 for depleted group). mRNA expression levels of Bmp4 and Fgf-2 were significantly decreased in vitamin D-depleted rats *versus* controls. Data presented are means ± SEM; *p < 0.05. **(C)** Protein expression of PCNA in *tibialis anterior* was analyzed by western blotting and quantified using Multi Gauge V3.2 software. Expression of the total amount of p38 was used to normalize protein loading between samples (n = 7 for control group and n = 9 for depleted group). The expression of PCNA protein decreased in old rats after dietary vitamin D depletion compared to control. A.U = arbitrary units. Data presented are means ± SEM; *p < 0.05.

Concerning genes related to cell proliferation, mRNA expression levels of Bmp4 (Figure 4A) and Fgf-2 (Figure 4B) were also significantly decreased by 39% and 31% in vitamin D-depleted rats *versus* controls (p < 0.05). These data were confirmed by the decreased expression of PCNA protein in old rats after dietary vitamin D depletion (-56%, p < 0.05, Figure 4C).

**Figure 5:**
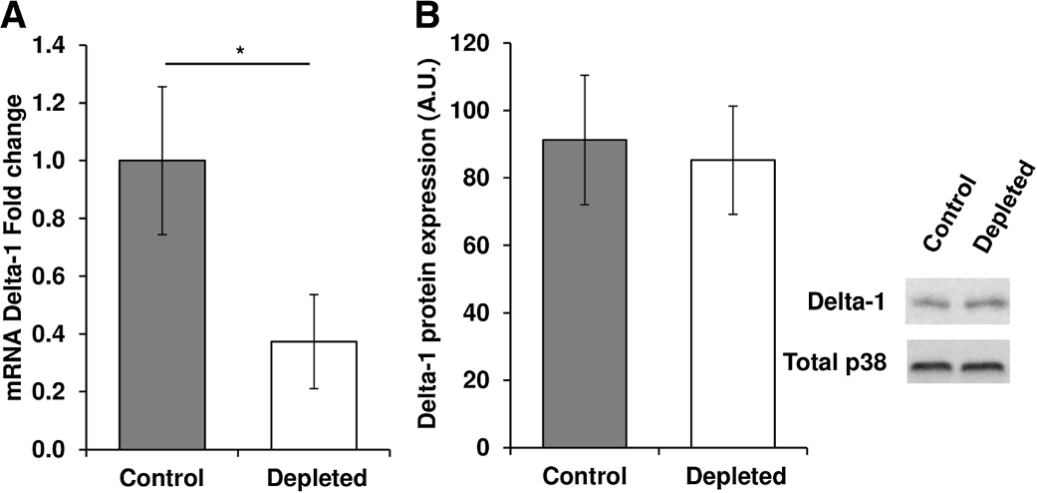
**Effect of vitamin D depletion on Delta-1 transcript levels and protein expression in old rats. (A)** mRNA expression of Delta-1 in *tibialis anterior* of control and depleted rats was analyzed using a RT^2^ Profiler Custom PCR Array following the manufacturer’s protocol. Delta-1 mRNA in control and vitamin D depleted samples was normalized using expression of Tbp as a housekeeping gene and was relative to control group according to the 2^-ΔΔCT^ method (n = 7 for control group and n = 9 for depleted group). Delta-1 mRNA level was reduced in the TA of vitamin D-depleted rats compared to controls. Data presented are means ± SEM; *p < 0.05. **(B)** Protein expression of Delta-1 was analyzed in *tibialis anterior* by western blotting and quantified using Multi Gauge V3.2 software. Expression of the total amount of p38 was used to normalize protein loading between samples (n = 7 for control group and n = 9 for depleted group). Delta-1 protein expression remained unchanged between the two groups. A.U = arbitrary units. Data presented are means ± SEM; *p < 0.05.

**Figure 6:**
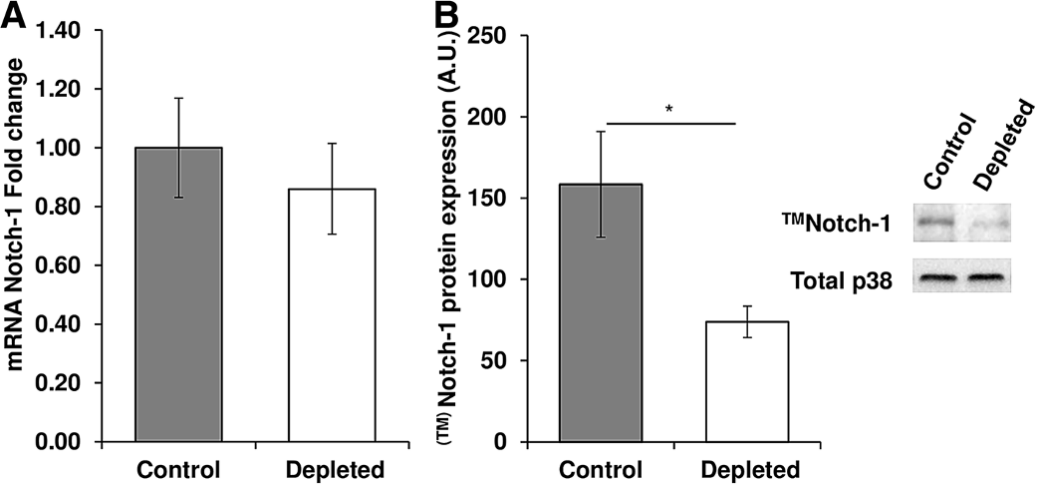
**Effect of vitamin D depletion on Notch-1 transcript levels and protein expression in old rats. (A)** mRNA expression of full length Notch-1 in *tibialis anterior* of control and depleted rats was analyzed using a RT^2^ Profiler Custom PCR Array following the manufacturer’s protocol. Full length Notch-1 mRNA in control and vitamin D depleted samples was normalized using expression of Tbp as a housekeeping gene and was relative to control group according to the 2^-ΔΔCT^ method (n = 7 for control group and n = 9 for depleted group). mRNA expression of full length Notch was unaffected by vitamin D depletion. Data presented are means ± SEM; *p < 0.05. **(B)** Protein expression of transmembrane fragment Notch-1 (^TM^Notch-1) was analyzed in *tibialis anterior* by western blotting and quantified using Multi Gauge V3.2 software. Expression of the total amount of p38 was used to normalize protein loading between samples (n = 7 for control group and n = 9 for depleted group). Protein expression of cleaved Notch (^TM^Notch) was decreased in old D-depleted rats compared to controls. A.U = arbitrary units. Data presented are means ± SEM; *p < 0.05.

**Figure 7:**
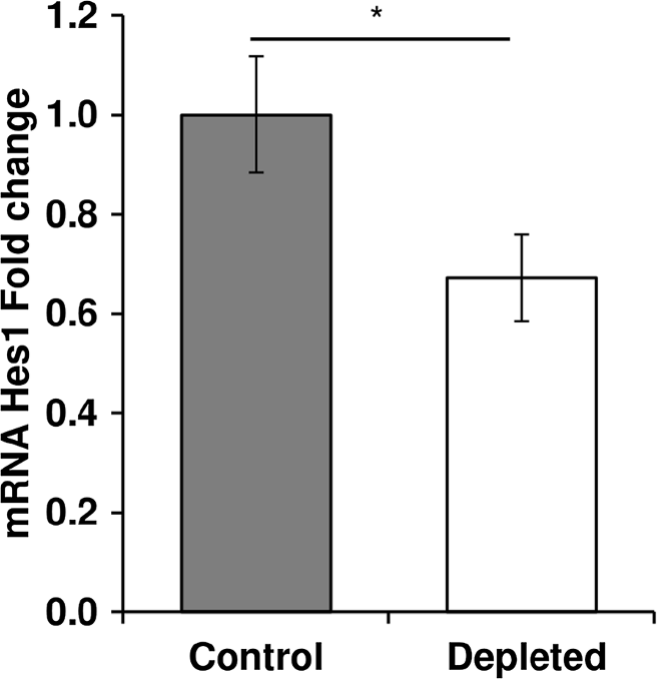
**Effect of vitamin D depletion on Hes1 gene expression in old rats.** mRNA expression of Hes1 in *tibialis anterior* of control and depleted rats was analyzed by using a RT^2^ Profiler Custom PCR Array following the manufacturer’s protocol. Hes1 mRNA in control and vitamin D depleted samples was normalized using expression of Tbp as a housekeeping gene and was relative to control group according to the 2^-ΔΔCT^ method (n = 7 for control group and n = 9 for depleted group). The expression of Hes1 was significantly reduced after vitamin D depletion in old rats. Data presented are means ± SEM; *p < 0.05.

The Notch pathway regulates skeletal muscle proliferation. Data from the PCR array showed that the expression of Notch intermediates was altered in the vitamin D-depleted group. Delta-1 mRNA level was significantly reduced by 63% in the TA of vitamin D-depleted rats compared to controls (Figure 5A). Unexpectedly, despite a significant reduction in its transcript level, Delta-1 protein expression remained unchanged between the two groups (control *vs*. depleted: 91.28 ± 19.23 *vs*. 85.27 ± 16.03, A.U., p = NS, Figure 5B). Although mRNA expression of full length Notch was unaffected by vitamin D depletion (control *vs*. depleted: 1 ± 0.17 *vs*. 0.86 ± 0.15, p = NS Figure 6A), protein expression of cleaved Notch (^TM^Notch) was decreased in old D-depleted rats compared to controls (-53%, p < 0.05, Figure 6B). Following the activation of the Notch pathway, the intracellular domain of the Notch receptor translocates to the nucleus where it acts as a transcription factor to promote the transcription of its target genes. Therefore, we analysed the mRNA levels of its target Hes1. In addition, as already shown, the variation of the mRNA expression of Notch targets reflects the Notch pathway activity [21, 24]. We observed that the expression of Hes1 was significantly reduced after vitamin D depletion in old rats (-35%, p < 0.05, Figure 7).

## Discussion

Sarcopenia is defined as the involuntary loss of muscle mass and strength with ageing [1]. The diminished ability of aged muscle to self-repair is a key driver of this process [4, 6, 28, 57], whether in situations of overt injury as in small daily damages not perceived *via* pain or altered contractile function [5]. The regulatory activity of the Notch pathway ―a key factor of muscle development and regeneration [17, 58]― also decreases with age and may contribute to muscle atrophy [12, 14]. Besides these endogenous regulatory pathways, environmental factors also play a key role in muscle regeneration [28]. Vitamin D could be central to maintained muscle mass due to its known effects on skeletal muscle [34]. Vitamin D status is positively correlated to muscle strength/function [34]. Older populations commonly develop vitamin D deficiency, causing muscle weakness [42]. Here, we tested the hypothesis that vitamin D deficiency contributes to the age-related muscle atrophy, due at least in part to a reduced Notch pathway activity. At the end the experimental period, blood 25(OH)D concentration had fallen by 75% in vitamin D-depleted rats. Despite similar food intake levels, average body weight increased in D-depleted rats compared to controls. Furthermore, while body fat mass increased in D-depleted rats, percent lean mass decreased. In accordance with this decreased fat free mass, D-depleted old rats showed a significant reduction in muscle mass, particularly in type II muscle mass. Interestingly, age-related muscle atrophy is characterized by a loss of muscle fibers, notably type II fibers [59, 60]. In this context, our study focused on TA, as it is mainly composed of type II fibers [61]. Our results on muscle mass are consistent with studies showing that vitamin D status is inversely correlated with body fat mass [62, 63] and that muscle is a direct target of vitamin D [34, 64]. Vitamin D potentiates protein synthesis in C2C12 following leucine and insulin treatment and regulates muscle contractile function [40, 41, 53]. Vitamin D is required for normal skeletal muscle development, and it promotes skeletal muscle regeneration following injury in adulthood [39, 65]. Finally, hypovitaminosis D develops with ageing and is linked to muscle weakness which increases the risks of falls [66]. All data from literature show that vitamin D is essential in regulating skeletal muscle structure and function. Interestingly, Schubert et al. have demonstrated that hypophosphatemia is responsible for skeletal muscle weakness in vitamin-D deficient rat [43], demonstrating that vitamin D effects on muscle could also be dependent of other factors. Vitamin D is known to regulate phosphocalcic homeostasis [67]. In our study, vitamin D depletion had no effect on serum phosphorus and calcium levels indicating that muscle atrophy was not a consequence of a phosphocalcic imbalance but was likely due to the vitamin D deficiency.

A logical effect of the reduction in body vitamin D status is that the expression of its receptor VDR was, as expected, down-regulated in the skeletal muscle of old D-depleted rats. Ceglia et al. [68] had previously shown that vitamin D_3_ supplementation increases VDR expression and fiber size in skeletal muscle of elderly women [68]. Furthermore, vitamin D, and specifically its active form 1,25(OH)_2_D, auto-regulates the expression of the VDR gene through intronic and upstream enhancers [53, 69]. Taken together, these results highlight that vitamin D depletion for 9 months provoked a vitamin D deficiency in old rats and subsequently generated morphological and molecular changes related to hypovitaminosis D.

In order to understand why muscle mass is reduced in D-depleted rats, we ran a PCR array to study the expression of genes related to autocrine signalling, apoptosis, metabolism, anabolism regulation, myogenesis, notch pathway, cell proliferation and cell structure and function. Except for the autocrine signalling, the expression of at least one gene from each listed clusters was down regulated in the vitamin D depleted group. These results highlighted that vitamin D deficiency displays a large variety of metabolic and functional changes in skeletal muscle. Thus, the effects of vitamin D depletion on these pathways need to be further investigated in skeletal muscle. In our study, we choose to focus on cell proliferation and Notch pathway. Previous studies have demonstrated that Notch signalling, muscle cell proliferation and vitamin D status are impaired in older people, and that vitamin D modulates muscle cell proliferation and stimulates regeneration. We found that the expressions of key genes related to the regulation of cell proliferation, particularly Bmp-4 and Fgf-2, were down-regulated with vitamin D depletion. The BMP proteins are known to be involved in myogenesis [70, 71]. In a murine C2C12 myoblast cell line, Terada et al. [45] found that Bmp-4 regulates myoblast proliferation [45]. On Fgf-2, studies using cultures of muscle cells or fibers in which SC were maintained in their *in situ* position, i.e. under the fiber basement membrane, have established that Fgf-2 enhances proliferative rate or the number of SC [46, 72]. Interestingly, previous studies have shown that 1,25-dihydroxyvitamin D modulates Bmp-4 and FgF-2 expression [47, 48].

As Bmp4 and Fgf-2 regulates muscle cell proliferation, we aimed to confirm that proliferation state where diminished in TA muscle of D-depleted rats. PCNA protein expression was decreased in vitamin D-depleted old rats compared to controls. PCNA reflects the proliferative activity, particularly in regenerating skeletal muscle [73–75]. The reduction in the muscle proliferative capacity of depleted old rats is consistent with a previous study showing that vitamin D stimulates muscle cell proliferation in rat skeletal muscle [39]. However, *in vitro* studies have established that 1,25-dihydroxyvitamin D treatment of a C2C12 muscle cell line inhibited cell proliferation [38, 76], whereas others have concluded that this hormone stimulates muscle proliferation [35, 37]. These works demonstrate that i) *in vitro*, the ability of vitamin D to modulate cell proliferation depends on cell culture conditions, and that ii) *in vivo* models make it possible to account for intrinsic and extrinsic factors, both of which can influence cell proliferation [27, 77, 78].

Here, we have shown that hypovitaminosis D aggravates the muscle atrophy in old rats as the expressions of key markers modulating muscle cell proliferation are down-regulated. While no experimental injury was induced in our model, discrete episodes of repair and regeneration occur nonetheless due to small daily damages. SC intervene in such situations although their activation is not necessarily detectable [5]. Here we speculate that vitamin D deficiency could result in a poor recruitment of SC into their proliferating state, due at least in part to a down-regulation of Fgf2, making them ready for programmed differentiation, as Bmp-4 is down-regulated. To validate this hypothesis, a model of vitamin D-depleted old rats undergoing muscle acute injury will allow evaluating the effect of vitamin D on SC activation using specific markers as Pax7 and MyoD or Pax7 and PCNA.

The Notch pathway is involved in SC proliferation [14, 79], and PCR arrays showed that the expression of some of the key markers of this pathway were modulated following vitamin D depletion. The mRNA expression of Delta-1, a Notch activator, was lowered in the vitamin D-depleted group whereas the expression of its protein remained unchanged between the two groups. This discrepancy could be due to a post-translational regulation to maintain in-cell Delta levels, as this protein can undergo recycling after its action on the Notch pathway [80]. Here, Notch mRNA expression was unaffected by vitamin D depletion in old rats whereas the expression of the cleaved Notch form (^TM^Notch) was reduced, reflecting a drop in activation of the Notch pathway. The down-regulation of ^TM^Notch expression in vitamin D-depleted old rats was not the consequence of a change in the expression of Notch pathway activator Delta-1, as previously demonstrated by Conboy et al. [13, 27]. The modulation of ^TM^Notch expression in depleted old rats was probably not associated to down-regulation of Notch receptor, as Notch transcript levels remained stable between vitamin D-depleted and control rats. However, Notch receptor, like Delta-1, can undergo recycling after their activation [81]. Therefore, the observed down-regulation of ^TM^Notch expression in vitamin D-depleted old rats may be due to a decrease in the proteolytic processing of Notch receptor, involving an ADAM protease and/or the γ-secretase complex [18–20]. Thus more investigations are needed to evaluate if ADAM or γ-secretase complex could be two targets of vitamin D, providing a new possible explanation of the down-regulation of ^TM^Notch expression following vitamin D depletion. The reduced activity of the Notch pathway in vitamin D-depleted rats was further confirmed by the decreased Hes1 mRNA expression in this same D-depleted group. Once the pathway is activated, Notch receptor is cleaved and its intracellular domain acts as a transcription factor to induce Hes1 gene expression. Hence, any up-regulation of Hes1 expression is related to activation of the Notch pathway, and inversely, Hes1 mRNA down-regulation reflects the reduced activity of the Notch signalling pathway [21, 24, 80].

The choice of an animal model in which no overt injury was done provided to us the possibility for studying Notch pathway signalling in the context of repair and regeneration of daily small damages which contributes to age-related muscle atrophy [5, 21]. However, to fully evaluate the effect of vitamin D deficiency on muscle regeneration with aging, future investigations using old and young animal models of regeneration will allow us to investigate the impact of vitamin D on a high regeneration process. The present study has raised new hypotheses. First, since hypovitaminose D affects muscle mass, the severity of muscle damages following acute injury is likely to be increased in vitamin D depleted old rats than in non-depleted animals. Second, it is possible that the efficiency of muscle recovery after injury is slower in vitamin D depleted old rats than in control old or vitamin D-depleted young rats. Third, the rate of satellite cells recruitment in old animals with vitamin D depletion is likely altered. Future investigations should be done in order to answer these questions.

## Conclusion

The present work has shown that vitamin D depletion for 9 months efficiently induced vitamin D deficiency in old rats and subsequently generated morphological and molecular changes related to hypovitaminosis D. Vitamin D depletion induces skeletal muscle atrophy in old rats through a reduction in the proliferative ability and in Notch pathway activity in skeletal muscle. The activation of Notch pathway activity is highly implicated in the mechanism of muscle regeneration, vitamin D deficiency could further aggravate the age-related impaired capacity of muscle to regenerate. These findings strongly suggest that vitamin D status needs to be controlled in elderly people to maintain muscle mass.

## Electronic supplementary material

Additional file 1: **Gene expression levels in** ***tibialis anterior*** **of control and vitamin D-depleted old rats.** Expression levels of genes known to regulate apoptosis, autocrine signalling, metabolism, anabolism, myogenesis, Notch pathway, cell proliferation, cell structure and function were analyzed in *tibialis anterior* of control and vitamin D-depleted old rats using a RT2 profiler custom PCR array. Data presented are means ± SEM. *: p < 0.05, **: p < 0.01 versus controls. (XLSX 11 KB)

## Abbreviations

25(OH) D: 25-hydroxyvitamin D
Bmp4: Bone morphogenetic protein 4
Fgf-2: Fibroblast growth factor 2
FM: Fat mass
Hes1: Hairy and enhancer of split-1
LM: Lean mass
^TM^Notch-1: Transmembrane fragment Notch-1
PCNA: Proliferating cell nuclear antigen
SC: Satellite cell
TA: Tibialis anterior
VDR: Vitamin D receptor.
